# The Social Drama of Mental Health Professionals who are also Former Mental Health Service Users

**DOI:** 10.1007/s11013-025-09955-y

**Published:** 2025-11-02

**Authors:** Karina Stjernegaard, Lene Lauge Berring, Sidse Marie Arnfred, David Crepaz-Keay, Niels Buus

**Affiliations:** 1 Psychiatric Research Unit, Psychiatry Region Zealand, Fælledvej 6, 4200 Slagelse, Denmark; 2 PsykInfo, Psychiatry Region Zealand, Københavnsvej 26F, 1, 4000 Roskilde, Denmark; 3 Department of Regional Health Research, University of Southern Denmark, Campusvej 55, 5230 Odense, Denmark; 4 University College Absalon, Trekroner Forskerpark 4, 4000 Roskilde, Denmark; 5 Department of Clinical Medicine, University of Copenhagen, Blegdamsvej 3B, 2200 Copenhagen, Denmark; 6 Mental Health Foundation, Studio 2, 197 Long Lane, London, SE1 4PD UK; 7 Department of Public Health, Aarhus University, Dalgas Avenue 4, 8000 Aarhus C, Denmark; 8 Monash University, 10 Chancellors Walk, Wellington Road, Clayton, VIC 3800 Australia

**Keywords:** Lived experience, Mental health professional, Mental health services, Social drama, Liminality

## Abstract

Studies indicate that the lived experience of being a mental health service user is common among mental health professionals. However, little is known about how such experiences may influence clinical practice. Through interviews and diary notes from fourteen Danish mental health professionals, we explored how these experiences become part of everyday practices. Data were coded and analyzed following an abductive process incorporating the theory of social drama by Victor Turner. We propose a conceptual model of the transitional challenges faced by these professionals within the current social order of Danish mental health services. For some, the lived experience disturbed the social order to such a degree that they questioned their employment; for others, lived experience was either shared verbally or concealed from service users and/or colleagues in ways that did not disturb the social order significantly. The proposed conceptual model points to dichotomies of service users versus professionals and of madness versus normalcy as evident discursive practices within mental health services that do not favor mental health professionals drawing on their lived experience.

## Introduction

Studies indicate that having lived experience of mental health challenges and trauma can be a motivational factor for working within mental health services (Barnett, 2007; Bryce et al., 2023; Farooq et al., 2014; Ong et al., 2017). However, it is uncertain how many mental health professionals have such experiences. For instance, the UK-based project “Hidden Talents” revealed that 53% of mental health professionals have lived experience of mental health challenges (Morgan & Lawson, 2015). A US survey of 101 mental health professionals revealed that 75% had a history of “mental illness” (Harris et al., 2016). A recent German study found that more than 84% of mental health professionals (n=215) had experienced mental health crises, and that several of them had used mental health services (von Peter et al., 2024). In addition to recognizing that some professionals have these experiences, studies have examined lived experience in different professions, e.g., social workers (Holley et al., 2024), art therapists (Huet & Holttum, 2016), counselors and psychotherapists (Cleary & Armour, 2022), psychiatrists (Boomsma-van Holten et al., 2023; Karbouniaris et al., 2023), and nurses (Oates et al., 2017, 2018). Other studies have explored lived experience in mixed groups of professionals, e.g., “researchers and providers” (Gupta et al., 2023), “psychiatrists, psychologists and others” (Frese et al., 2009), and “different professional disciplines” (Richards et al., 2016). However, across the studies, the concept of “lived experience” covers vastly different experiences of mental health challenges, concerns, crises, trauma, and of being a service user.

A growing number of professionals with lived experiences as service users are publishing their accounts as autoethnographies (e.g., Fisher, 2023; Freisen, 2022; Vierthaler & Elliott, 2020). Vierthaler, a trained psychologist, shared that she was advised not to speak publicly about her experiences if she wanted to be a successful psychologist. She emphasizes the struggle of a person with lived experience as a service user and whether that person may also be perceived as an eligible professional by colleagues (Vierthaler & Elliott, 2020). Fisher (2023) emphasized the need for a reconstruction of the values connected to the role of mental health professionals with lived experience, e.g., being between the social roles of a nurse and a service user and not knowing if she belongs in the “staff office or the patient’s waiting room?” (Fisher, 2023, p. 881). Venturing back to the late 1990s, Brandon (1999) vividly captured the complexity of not belonging to any social role:

People like us can get rejected by both user and professional camps for different reasons – by professionals because of our unreliability and instability; by fellow patients because we are somehow tainted because we’ve been on the wrong side of the couch. (Brandon, 1999, p. 322)

The perceptions of mental health professionals who share their lived experience and the ways in which they perceive themselves are influenced by local culture (King et al., 2020). Some are perceived as *impaired professional*s (Telepak, 2010), whereby their professional capacity is questioned due to their lived experience. Some perceive themselves as *wounded healers* (Conchar & Repper, 2014; Zerubavel & Wright, 2012), whereby experiences of mental health challenges are perceived as a resource within services. Others perceive themselves as *prosumers* (professional consumers), underlining reciprocity with service users while maintaining a professional role (Manos, 1993; Tsai, 2002). Still, others perceive themselves as *survivor-therapists*, referring to professionals who experienced human rights violations as service users (Adame, 2011). Either way, a recent review (Karbouniaris et al., 2020) concluded that understanding the values of lived experience among mental health professionals is still in its early stages of development.

Some mental health professionals with lived experience are part of the growing workforce of peer support workers (Bellamy et al., 2017; Davidson et al., 2012; Gillard & Holley, 2014; Repper & Carter, 2011; Shalaby et al., 2020; White et al., 2020). Peer support workers are a specific profession, where people are employed specifically due to their lived experience of mental health challenges, their experiences of being in recovery, and in some cases their experiences of being users of mental health services (Shalaby et al., 2020). The primary task of peer support workers is to share their lived experiences to support others, as inspired by the consumer/survivor/ex-patient/service user movements of the 1970s (Bellamy et al., 2017; Shalaby et al., 2020; Watson, 2019). Consequently, peer support workers are challenging biomedical understandings of mental health challenges (Frieh, 2024) as well as traditional objective expertise by insisting on the relevance of subjective experiential knowledge (Oborn et al., 2019). Even though peer support workers face challenges of not being acknowledged within the collective workforce (Charles et al., 2020; Cooper et al., 2024; Moran et al., 2013), it is suggested that peer support workers can facilitate a more inclusive workplace where all employees can share lived experiences with mental health challenges (Byrne et al., 2022)**.**

Participants in our study are not peer support workers, but healthcare professionals with lived experiences as service users, and we try to grasp how they incorporate their lived experiences into clinical practice. The study, therefore, examines the process of integrating lived experiences of mental distress into the role of a mental health professional, not the participants’ individual experiences of being in recovery even though these processes might influence each other. In the current context, we define “integration” as sharing experiences verbally or altering practices based on personally experienced incidents in ways that disturb norms within mental health services.

## When Having Lived Experiences Manifests as a Social Drama

We make use of Turner’s (1967, 1969, 1974) concept of “social drama,” which refers to collective action characterized by conflict, heightened emotions, and a temporary breakdown of social order. Social order refers to the socially structured aspects of a society or group, including norm-governed social rules, roles, hierarchies, and cultural values (1969). Social dramas manifest when social order is disrupted by conflict arising within groups or individuals, leading to anti-structure. The oscillation between structure and anti-structure is Turner's analytical take on how rituals function like a dynamic social drama because they are symbolic dynamic actions to communicate meaning.

Social drama encompasses a series of four stages: breach, crisis, redressive action, and reintegration/schizogenesis (Turner, 1974). (1) A *breach* is an event or action in which a group or an individual disrupts the social order that exists within a system of social relations. (2) Following the breach is a period of heightened tension and conflict unless the breach can be sealed off quickly. Turner described this *crisis* as a turning point. (3) The ongoing crisis leads to the stage of *redressive action*. Members of the disturbed social system will use redressive mechanisms to try to restore order. If redress fails, the crisis will prevail, and the society, group, or individual may fall “betwixt and between” (Turner, 1967, pp. 93–111) social roles in a liminal state. In the liminal state, the “liminal persona” is “no longer classified and not yet classified” (Turner, 1967, p. 96). This leaves room for transformation and possibility but is also a state in which the liminal persona is stripped of their social status, leaving the liminal persona structurally invisible. The liminal personas may engage in *communitas*, which is a sense of solidarity, equality, and close connection between individuals during the liminal period (Turner, 1967). Communitas can be experienced as a physical interactional connection or an inward experience of existential connection (Turner, 1969). (4) The final stage is for the social drama to end with the *reintegration* of the disturbed social group or individual into the social order (thereby upholding the social order) or with a recognition of the crisis being insoluble, resulting in *schismogenesis* or breakdown. The latter creates a rupture or division within a social group or society.

In this article, we perceive the struggle of a mental health professional who tries to integrate lived experiences into everyday practices as a *social drama*. The social drama takes place within the social order of Danish mental health services. These services are generally bio-medically oriented, with pharmaceutical treatment of most service users, and a relatively high rate of coercive measures, which has not decreased for the last decade (Sundhedsstyrelsen, 2024). Like many Western countries, Danish mental health services are struggling with the recruitment and retainment of professionals and equitable accessibility to services. Using the four stages of Turner’s social drama as a framework for understanding social conflict within this social order and its possible resolution allows us to perceive the actions of the mental health professionals as situated within a social order that conflicts with the use of lived experiences.

Applying this theory also emphasizes the lack of community among these mental health professionals and, hereby, the lack of possibility to engage in physical supportive connections with others who shared similar experiences. The participants shared common ideological values significant to communitas: they strived to be included as *equal* mental health professionals within their different social contexts and did not refer to each other with regard to specific professions or other hierarchical positions. In this sense, they were connected in *existential communitas* in their shared experiences of exclusion, or fear of exclusion, and in their attempts to solve their crisis in different ways, even though they did not interact directly with other mental health professionals who were in a similar liminal state. This type of communitas is, however, fleeting since the experienced shared human togetherness, that is, communitas, is in this case existential and not validated through physical or verbal interaction.

Finally, the theory of social drama not only elucidates the dynamics of conflict and possible resolution but also provides insight into the transformative potential of the integration of lived experiences and professionalism in mental health practices—and whether this integration is even possible within the current social order.

## Method

We used an abductive approach to analysis and data collection (Timmermans & Tavory, 2022). Data consisted of interviews and diary notes from current mental health professionals with lived experiences as service users.

### Sampling and Participants

The study included fourteen Danish mental health professionals currently working in mental health services. Participants were recruited using purposive sampling (Patton, 2002) via posts on social media (Facebook and LinkedIn) and local workplace newsletters. One participant was recruited through snowball sampling when a participant introduced a colleague to the project.

Participants were included according to the following two criteria: (1) current employment as a mental health professional with daily interactions with mental health service users and (2) lived experiences as a mental health service user. Mental health professionals working as peer support workers and similar were excluded. The participants included in the study cover a diverse set of professions and sectors (see Table 1).

**Table 1:** Participants, mental health professionals (*N*=14)

Participant pseudonym	Sex	Healthcare profession	Workplace	Years of experience within mental health services	Diary notes	Shares lived experiences with service users	Shared lived experience with management and colleagues	Last contact with services as a service user (youth/ adulthood/recently)	Participated in second interview
Nynne	F	Social Educator	Inpatient	5	Y	Some	Some	Recently	
Bodil	F	Psychiatric nurse	Inpatient	5	Y	Some	Some	Recently	X
Louise	F	Enrolled nurse	Inpatient	22	Y	Some	Some	Adult	
Ditte	F	Psychiatric nurse	Outpatient	7	Y	Some	Some	Adult	X
Christina	F	Psychiatric nurse	Outpatient	4	Y	Some	Yes	Adult	X
Jane	F	Psychiatric nurse	Outpatient	24	Y	No	Some	Adult	
Clara	F	Psychiatric nurse	Inpatient	4	Y	Some	Some	Adult	
Victoria	F	Psychiatrist	Outpatient	14	N	No	No	Adult	X
Line	F	Enrolled nurse	Inpatient	15	Y	Some	Yes	Adult	
Rune	M	Social Educator	Inpatient and supported housing	9	N	No	No	Adult	
Jacob	M	Psychiatrist	Inpatient	3	N	No	Some	Adult	
Michael	M	Enrolled nurse	Inpatient	6	N	Some	Yes	Adult	
Tanja	F	Psychiatric nurse	Inpatient	7	Y	Some	Some	Adult	X
Mette	F	Occupational therapist	Supported housing	5	Y	Some	Yes	Adult	

### Interviews

Between December 2022 and September 2023, the first author (KS) interviewed the fourteen mental health professionals using a semi-structured interview guide. Due to expected concerns regarding stigma, the participants were asked to choose the setting of the interview based on where they felt safe to share their lived experiences. The interviews were, for instance, conducted at local cafés, at the participant’s workplace, in the participant’s home, or online via Teams.

### Diaries

Participants submitted event contingent diary notes (Nezlek, 2012) after the interviews, describing interactions in which they felt influenced by their lived experiences. Four participants did not return their diaries (see Table 1) due to (1) not feeling comfortable with the written response format; (2) frustrations about management not allowing them to share their lived experiences, which made the diaries meaningless; or (3) a high workload.

### Analytical Approach

All interviews were audio-recorded and transcribed verbatim by the first author (KS) or an assistant. The diary notes were transcribed and anonymized by the first author (KS). The initial open coding was kept close to the data. Codes and index cases were discussed within the collective group of authors (KS, LLB, SMA, DC, and NB) and with an advisory board consisting of current service users, peer support workers, mental health professionals with lived experiences of mental health challenges, researchers, consultants, and managers of mental health service units. Memos were written in parallel with the coding process, during which the initial codes were sorted and compared. Moving on to focused coding, we explored variations of *how* and *with what consequences* the participants’ practices were influenced by their lived experiences as service users. Codes and categories indicated the participants’ concerns and attitudes toward their daily practices and their encounters when sharing their lived experiences, their experiences with injustice, and their attitudes toward the expected ways of being professionals. To explore variations within categories more data were collected by re-interviewing five of the participants (see Table 1) from November 2023 to February 2024.

According to the abductive analytic approach by Timmermans and Tavory (2022), the previous theoretical knowledge of the researchers has the potential to serve as a lens through which data can be perceived in different ways, thereby facilitating surprises through the recursive movement between inductive and deductive reasoning. Timmermans and Tavory (Tavory & Timmermans, 2019; Timmermans & Tavory, 2012) have challenged the use of the inductive approach, arguing that novel theories need to be connected to the body of theory in which the research is rooted. During the process of analysis and theory building, the notion of *social drama* by Victor Turner was introduced due to the nature of the emerging categories of “not belonging” and “sharing lived experiences to fit practice” after we had scrutinized and rejected other possible theories. By revisiting data while reading theory (as exemplified by Timmermans, 1998, 1999), the categories gained theoretical traction with regard to the disruption or upholding of social order that was at play for the participants in a state of “not belonging.” The abductive approach, which included the initial open coding, the later introduction of the theory, and going back and forth between theory and data, led to the proposed conceptual model of the social drama that manifests when lived experiences become part of the everyday practices of these mental health professionals within mental health services.

## Results

We propose a conceptual model of the process of mental health professionals with lived experiences who in distinctly different ways tried to integrate their lived experiences into the social order of mental health services. The conceptual model includes two different ways in which these professionals attempted to solve their crisis of not fully belonging: by integrating lived experiences into the role of being an expert or by fighting for a new role as a mental health professional with lived experiences. These actions ended the social drama in both reintegration and breakdown (schismogenesis). See Model 1 for the conceptual model of the social drama.

**Model 1:**
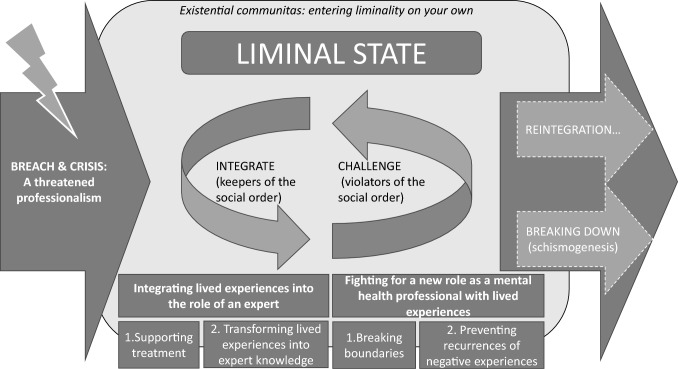
Stages of the social drama

### Breach and Crisis: A Threatened Professionalism

The mental health professionals included in this study broke the social order within mental health services by virtue of being *both* professionals *and* a person with lived experience as a mental health service user. The participants stated that service users and mental health professionals are considered distinctly different, non-overlapping groups within services, which were a source of conflict, worry, and potential transformation. One of the participants described a “Tarzan culture.” This is a common expression in Denmark, referring to professionals who are expected to be infallible and invulnerable rather than show emotions or express vulnerabilities.

The crisis following this breach was experienced by the participants when they could no longer uphold the rationality of a “sane” mental health professional. They experienced the crisis as having difficulties integrating their experiences into the role of a professional in different ways. The crisis was expressed by the participants when interacting with service users, colleagues, and management, as well as in the overall experience of being a mental health professional within the social order of mental health services.

Ditte was a mental health nurse who had worked at an outpatient treatment center for 7 years. She had lived experiences as a service user from her early youth and was still struggling with a feeling of being different from everyone else. She had experiences from both inpatient and outpatient group therapy settings as a service user. Meeting other service users was particularly meaningful for her during the time when she was receiving treatment. Ditte worried about sharing her experiences with service users and professionals:

That’s the thing that’s always at the back of your mind, isn’t it. They [service users] shouldn’t think I’m weak (…) I’m constantly sitting with the thought that someone now thinks that I’m not professional. (Ditte, mental health nurse, interview data)

What characterized Ditte’s crisis and entry into a liminal state was that she worried about not being recognized as a professional by service users, colleagues, and management. She did not perceive herself as weak but worried that others might; therefore, she tried to uphold the demeanor of a non-weak professional, even though she believed that she had a lot in common with the experiences of the current service users.

Clara, a mental health nurse who had worked at a ward for 4 years, experienced the breach and following crisis in a different way. She entered mental health services as a service user in her early teenage years. As a service user, she had confidence in the professional assessments of her challenges, even though she stated she was wrongly diagnosed and wrongly medicated for years. Clara was now in the position of carrying out the assessments as a mental health professional. Clara’s crisis emerged when she was challenged by service users’ perceptions of her when she shared her lived experiences of losing a parent at an early age or shared that she was living with recurring suicidal thoughts:

I think that the relationship [with the service user] after [sharing lived experiences] was different. If I call the relationship equal, then it sounds positive, but by me sharing my lived experiences, there was less recognition of me as an authority (…) the relationship or my professional [role] had broken a little bit. (Clara, mental health nurse, interview data)

Clara’s case exemplifies a crisis due to the service user’s reaction conflicting with the rationale of the mental health professional as an authority and expert. The “equal relationship” was perceived by Clara as a negative separation from her group of “sane” colleagues. Her experiences of this disruption of the social order made her question whether or how she could integrate her lived experiences into her role as a professional.

For both Ditte and Clara, the turning point that upheld the crisis was when they, as mental health professionals with lived experiences, were faced with the decision to share their experiences and thereby potentially risk their professional positions. All the participants entered the liminal state when trying to resolve this crisis of how their lived experiences could become part of their professional roles and the social order within mental health services.

What characterized the participants’ crisis and entry into the liminal state was not only feelings of worry and conflict but also profound loneliness. The participants did not feel connected to colleagues with similar experiences (because lived experiences were rarely shared), and therefore, they entered the liminal state without belonging to a supportive group. They acknowledged and expressed that there was a group “out there” to be part of and felt as though they belonged to this group—even though the group was not yet structured.

### Integrating Lived Experiences into the Role of an Expert

The participants had an urge to disturb the social order due to their own experiences as service users. When they shared their lived experiences or changed their practices due to the influence of their experiences, the decision to do so was based on intentions of normalizing mental distress, supporting hope, and reducing experiences of stigmatization.

However, the social order significantly influenced how they solved their crisis and their actions in the liminal state. The social order included the notion of being an expert, of having the service user follow treatment, of upholding authority, and of being a team player. This was expressed in two distinct ways by the participants: by sharing lived experiences to support treatment or by upholding a professional role by transforming experiential knowledge into expert knowledge.

#### Sharing Lived Experiences to Support Treatment

In the liminal state, the participants made efforts to resolve the crisis of a threatened professionalism. One applied way of doing so was by sharing lived experiences in ways that complied with social order, as opposed to challenging it.

Bodil was a mental health nurse who had worked on a ward for 5 years. She experienced mental distress in her early childhood and perceived herself as “genetically dispositioned” to periods of “depression,” the most recent of which was only a few years previously, while she worked as a nurse. For this reason, she booked regular sessions with a psychologist. She shared positive lived experiences of medication and overcoming “depression” with service users. She did not, however, share negative experiences of her family background or specific diagnosis. She believed that having lived experiences made her a better nurse with “dual competence” since she could relate to the service users’ worries, as she exemplified in a diary note:

He [a service user] was very ambivalent about trying medication, skeptical, and afraid. I chose to tell my own story briefly about having lived with depression all my young life and tried to fix it myself but could not quite reach the goal. It was only when I finally dared to try medication that I really got better. That you can do so much yourself, but some chemistry in the brain you may need medical help for. I did not plan on saying anything but thought it fit the conversation as something that might give him a positive insight to take that next step. We had a good connection. He was happy to hear my story and found meaning in it. This led to several conversations about medicine and in the end, he tried it out. (Bodil, mental health nurse, diary note)

Bodil reflected on this diary note during a subsequent interview, when she was asked about her reflections on sharing lived experiences when a service user is ambivalent about the treatment proposed by the treatment team:

There are many [service users] who have heard a lot of bad things about medication. And I think that being mirrored by someone within the system [helps]. So, they know that ‘it’s not about her saying this because she represents psychiatry, that I must take medicine. She also says it because she can understand that I am worried’ (…) That’s the framework we have. So, I can’t go against the psychiatrist, for example. (Bodil, mental health nurse, interview data)

Bodil shared her experiences of taking medication to mirror the service users’ worries in a way that combined her lived experiences with a biomedical understanding of the service users’ distress as being connected to “chemistry in the brain.” This transformed her experiences into a powerful tool for motivating the service user to follow the treatment plan, collaborate, and start medication. She did this not only because she had positive experiences with medication, but also because she had to fit into the social order as a proper mental health nurse who supports the decisions of the team—and more specifically, the psychiatrist. Even though Bodil argued that she did not want to only “represent psychiatry,” she ended up upholding the decision of the psychiatrist instead of supporting the worries of the service user, thereby reproducing social order as an expert mental health professional.

This exemplifies an effort to *integrate* lived experiences into the social order and thereby not violate the social order. By doing so, these participants integrated their lived experiences into their everyday practices on the premise of the current social order, thereby becoming *keepers of the social order*. In this respect, the liminal state is characterized by the participants’ attempts to transform their lived experiences from idiographic experiences to generally relevant experiences that support the current biomedical understanding of mental health distress, whereby the lived experiences emphasize the mental health professional’s position as an expert.

#### Upholding a Professional Role by Transforming Lived Experiences into Expert Knowledge

Redressive mechanisms reflected colleagues’ and management’s expectations of the participants to remain professional by not basing their practices on their lived experiences. One reaction to these mechanisms was integrating lived experiences into everyday decisions concerning treatment in a more subtle way. This was exemplified by Rune, who camouflaged his lived experiences when talking to colleagues.

Rune was a social educator with 9 years of experience. He worked in both inpatient and outpatient settings. Rune’s lived experience dated back to his youth when, during high school, he was admitted to a mental health hospital. In the following years, Rune spent several years within the mental health system in wards, outpatient treatment centers, and supported housing. He vividly expressed experiences of stigmatization by mental health professionals when talking about his experiences of being a service user and how a diagnostic label seemed to follow him whenever he was in contact with healthcare and social services in general. Rune had never told service users, management, or colleagues about his lived experiences. Instead, he transformed his experiences into expert knowledge and thereby upheld his professional role:

I have a clear experience that my professionalism will be neglected the moment I tell people that I am mentally ill. (…) I am often very good at presenting my own ontological perspective as if it were an epistemological one. That is to say, I attribute to it a professionalism, or a starting point, or a knowledge that is more generalized. And that means, well, that what I know, I can present as if it were a more general understanding. (…) You must speak with authority to be heard. And authority is not talking about yourself. (Rune, social educator, interview data)

What Rune exemplifies is a mental health professional who does not challenge the social role of being an expert but challenges what expert knowledge is without anyone knowing from where his viewpoints originate. By doing so, the lived experiences are *integrated* into the role of an expert. In this way, Rune avoided stigma, but he also upheld the social order. How the lived experiences influence practice is still fixed within the current social order, leaving little room for Rune to express lived experiences that would significantly disrupt the decisions of the team.

These integration attempts imply an effort to solve the crisis of a threatened professionalism while not fundamentally disturbing the social order—which also makes these participants *keepers of the social order*. The redressive actions of the treatment teams kept the participants in place as experts.

### Fighting for a New Role as Mental Health Professionals with Lived Experiences

Some of the participants tried to mend the crisis of a threatened professionalism by breaking organizational boundaries or by influencing practice based on their own negative experiences of being a service user within mental health services. These liminal experiences were of professionals who were fighting to change the social order within mental health services in different ways.

#### Breaking Organizational Boundaries by Insisting on the Relevance of Lived Experiences

Most of the participants had told their managers about some of their lived experience as service users. Some were motivated to do so because they expected management to take any current challenges into account when making plans concerning workflow in the team. Others did so to disrupt the social order of mental health professionals not having anything in common with the people they were trying to support. Line was an example of the latter.

Line worked at different wards for 15 years as an enrolled nurse. She had experiences of being admitted to wards both in her youth and in recent years, and she had also received outpatient treatment. Her experiences included several suicide attempts, a drug overdose, and existential feelings of being different from everyone else. She emphasized that she had undergone electroshock treatment and that her diagnosis was meaningful for her as an explanatory framework for some of her past behavior and decisions. Her manager was privy to these experiences when Line signed her contract of employment. The manager, however, decided that Line’s lived experiences could not be accommodated as something to be shared with service users within the ward and could not be part of her practices as an enrolled nurse:

I was required not to say anything to the service users about my illness. (…) It just goes against everything in me. Not being honest (…) When a service user has a problem or something that hurts inside, I know what it feels like because I’ve experienced it a million times. I’d like to be allowed to say, ‘You know what, I know that feeling, it can get better’ (…) So I must admit that I have said something [to the service users] a few times. (Line, enrolled nurse, interview data)

Line rejected the social order expressed by management of what was expected of her as a professional by sharing her lived experiences with service users. The redressive action of not allowing Line to use her experiences failed: order was not restored, and Line’s crisis prevailed. Line chose to share her experiences with service users in secrecy. She did not share her experiences with suicide attempts or her diagnosis, but she emphasized that she shared “being in the same boat” as the current service users and that it is possible to get better because she had been “where they are.” Line was aware of the risk of losing her job but emphasized that her experiences were a source of motivation for her current employment, and she believed that sharing lived experiences supported service users in getting better.

Not following the social order, in Line’s case as instructed by her manager, placed these participants in a liminal state characterized by rule-breaking and riot—thus these participants became *violators of the social order*. These participants shared their lived experiences in secrecy hoping to challenge the service users’ feelings toward and expectations of the “sane” mental health professional, thereby challenging the social order indirectly, with an inherent and continuous risk of exposure.

#### Preventing Recurrences of Negative Experiences

All participants described both good and unsettling lived experiences of being service users within mental health services. In particular, the unsettling experiences influenced how the participants tried to mend the crisis of a threatened professionalism and how they reacted to the redressive actions. The unsettling lived experiences influenced some of the participants in their daily practices in their attempts to prevent recurrences of the negative experiences they faced as service users.

Jacob worked at a ward. He had a few years of experience as a psychiatrist. He had been admitted to a ward himself and described how he was wrongly assessed and diagnosed during his first admission in his early twenties due to his “untidy appearance.” He particularly remembered mental health professionals who wrote about him having drug use in several journal entries—even though Jacob had never used drugs. This specific experience influenced his trust in journal entries in general:

Just because my colleague has written that the service user does drugs, I don’t think that it’s automatically true. So, I ask the person again. So sometimes, my service users get upset, like: ‘I’ve talked about that!’ ‘But I want to be sure that I’ve understood it correctly and I also want to hear it from you, and I want to make sure that we give you the right treatment’. It is rooted in my own experience. I also felt that I was misunderstood. (Jacob, psychiatrist, interview data)

Based on Jacob’s lived experience as a service user, he believed that his colleagues may write information in the service user's journal that may not be true. Therefore, he continuously sought validation from the service users. Even though the service users sometimes reacted negatively toward his request for validation, he continued to do so as a way of challenging the notion that the mental health professionals’ assessments and observations always agree with the experiences of the service users. However, this practice may be perceived by his colleagues as mistrust and by service users as a practice that does not make sense, because they had to repeat their statements. The transformative potential of being influenced by one's own lived experiences as a mental health professional may therefore be misinterpreted—particularly when these professionals had not disclosed lived experiences to either service users or other professionals.

These participants did not give in to the social order. They were *violators of the social order*, but they were also still protecting themselves from stigma and from losing professional credibility by not sharing their lived experience within the team or with service users.

### Reintegration

Some of the participants found ways of ameliorating the crisis of a threatened professionalism by sharing their experiences in ways that adhered to the current social order. This was the case for Bodil, who only shared her positive lived experiences and only when they were aligned with the current treatment plan. Rune also exemplifies a reintegration that did not disrupt the current social order by camouflaging experiential knowledge as expert knowledge not explicitly linked to his lived experiences—experiences that the team did not know about. While mental health professionals like Rune and Bodil upheld the social order and were reintegrated, one might argue that their crises were not solved.

The crisis, and thus the social drama, was not resolved by a transformation of the social role of mental health professionals or by a change in the social order within services that could allow and support these professionals to share their stories and make changes in mental health services based on their lived experiences. None of the mental health professionals described reintegration at a level that enabled them to share all their lived experiences when it made sense to them in a professional context and within their roles as professionals. They all withheld some of their experiences. The lived experiences that were generally not shared explicitly were diagnoses associated with stigmatization, stories of abuse, and stories of being significantly mistreated within mental health services—services that the participants were currently employed in.

When a new social order was not realized, the participants were either trapped in the liminal state, still struggling to integrate their lived experiences into their professional roles and practices (and trying different methods of reacting to redressive actions), or the social drama ended in schismogenesis.

### Schismogenesis: Breaking Down

Some of the participants failed to solve the crisis of a threatened professionalism, which set them apart from their social groups, with the social drama ending in schismogenesis. An example was seen in the case of Christina.

Christina worked at an outpatient treatment center for 4 years as a mental health nurse. She had lived experience of being admitted to a ward and had received outpatient treatment some years previously. Her diagnosis was being reevaluated during the time of the interviews. As a service user, Christina felt threatened, misunderstood, and excessively observed. In Christina’s experience, the mental health professionals had a narrow focus on “fixing” her, rather than examining her social context and trauma. These experiences significantly influenced her daily practices as a nurse and how she perceived current treatment practices that she was expected to support. Christina reached out to the first author (KS) because she felt like “giving up” a few months after the first interview. She shared her experiences in the subsequent interview:

And there I just wanted to give up because I didn’t want to be there anymore. (...) [My colleagues say] that it is the service user who has challenges that we must try to solve. And I don’t think that's entirely correct, based on what I’ve experienced [refers to lived experiences of being a service user]. After all, the healthcare system can affect service users, but also society. (…) I’ve broken down several times. (Christina, mental health nurse, interview data)

For some of the participants, such as Christina, their lived experiences do not match the social order in which mental health challenges are for the most part perceived as challenges within an individual, as opposed to challenges that can be caused by social, structural, or environmental factors—or even arise from or worsen within mental health services. This was experienced to such a degree that these participants, being *violators of the social order*, questioned their employment. Some of the participating mental health professionals left services or strongly considered doing so by the end of the data collection period due to the division they felt between themselves and the broader social groups of mental health professionals.

## Discussion

The theory of social drama provided a meaningful framework for understanding and illustrating social conflict and change when these mental health professionals tried to integrate their lived experiences into their daily practices. However, the linearity of social drama, as portrayed in our conceptual model, may obscure the non-linear and possibly more chaotic nature of social conflict. Reintegration or schismogenesis may not follow a predictable pattern or capture all the nuances of the participants’ experiences. The linearity may also oversimplify that the complexity the participants were facing. We also acknowledge that using the theory of social drama as a framework may have portrayed our analysis as deductive rather than abductive—especially when the approach to sampling might be the reason why there are no outliers in the form of participants who did not experience a crisis when their lived experience influenced their professionalism and practice. Even though the theory of social drama was introduced late in the analysis, the use of the theory could overshadow elements of the iterative process and the fact that the conceptual model was initially data driven.

The theory of social drama seems to match the participants’ experiences of being in a state of “invisibility” and their experiences of transitional challenges. This is a different frame for perceiving and understanding the struggles of these mental health professionals than is applied in other studies. Mental health professionals with lived experiences of mental health challenges, crises, or of being mental health service users are often described as having dual or hybrid identities (see, for example, Adame et al., 2017; King et al., 2020). However, in the liminal state, these mental health professionals are not professionals and (former) service users—they are neither. They are “betwixt and between” social roles and must be classified anew within the social order. Perceiving the participants as solving a crisis within a social drama leaves room for emphasizing that mental health services may still struggle to understand how lived experiences and professionalism can coexist among mental health professionals who are not peer support workers, and that these mental health professionals may still be “structurally invisible” –as something that “ought not to be there!” (Turner, 1967, p. 98). Peer support workers may seem like the equivalent of the participants of our study. Peer support workers experience liminality in the sense of being “betwixt and between” social roles as service user, friend, and staff (Simpson et al., 2018); however, peer support workers are acknowledged as being in this, however, difficult position since it is known that their expertise is based on lived experiences rather than clinical training. Furthermore, peer support workers face different challenges. For example, peer support workers face challenges of drifting toward traditional practices rather than staying true to the values of peer support (Mead & MacNeil, 2006). The participants in our study are trained in traditional practices and have responsibilities attached to their professions. They are not transitioning from being a service user to becoming professionals like peer support workers (Tookey et al., 2018; Vandewalle et al., 2018). They are trying to incorporate lived experiences *into* professional roles and practices that have already been defined.

The majority of the participants shared some of their lived experiences of being service users with service users, management, and colleagues, and some of them stated that lived experience is appreciated. However, the participants in our study were very selective about which lived experiences they shared. Their colleagues perceived them as wounded healers when they shared experiences of anxiety or feelings of hopelessness, but not when they shared experiences of being in a ward or of hearing voices. Our results point to more complex social dynamics involving more than simply a decision of whether to share. Our proposed conceptual model points to a social order in which mental health professionals who insist on openly sharing their lived experiences are excluded if they do not do so within a narrow framework of being a professional expert. These mental health professionals with lived experience as mental health service users struggle to integrate their experiences into the “allowed ways of being a professional” (Richards et al., 2016) within the social order of mental health services. von Peter and Schulz (2018) describe the powerful boundaries between the “vulnerable” service users and “invulnerable” professionals in their analysis of category work within mental health services. They conclude that this dichotomous logic causes both mental health professionals and mental health service users to feel reduced. Experiences of “madness” do not seem to be compatible with the role of a mental health professional. With reference to a quote from a survivor-therapist, Adame (2014) argues that “there needs to be a place in society for madness” in which society embraces the differences and sometimes disturbing experiences of fellow human beings. This resembles the social construct of madness discussed by Foucault. According to Foucault (2001), madness is a concept shaped by societal norms, power dynamics, and historical context over time. By understanding madness as a social construct, Foucault takes a critical stance on fixed understandings of rationality and irrationality, emphasizing the influence of shifting social, political, and cultural forces. Foucault argued that the societal processes of separating the irrational from the rational—the sane members of society from the mad—are processes of deeming what is acceptable or deviant behavior within society. Foucault’s critique of the medical gaze (2003) and the societal construction of madness aligns with the objectives of Survivor Research (Russo & Sweeney, 2016; Sweeney et al., 2009) and Mad Studies (Beresford & Russo, 2021; LeFrancois et al., 2013; Lewis et al., 2024). These distinct fields of scholarship that build on the consumer/survivor/ex-patient/service user movements seek to disrupt traditional understandings of madness and mental health and elevate the lived experiences of the marginalized individuals within and outside mental health services who experience madness or distress. Within these movements, and the overall movements of people who either want to liberate themselves from psychiatry, psychiatric labeling, and who are seeking alternatives (often known as survivors), or people who argue for better choices and reduced stigma within the psychiatric system (often known as users or consumers). In both cases, the generation of knowledge from those who historically have been labeled mad is a way of challenging knowledge generation within the field of mental health (Hölling, 2001). Our conceptual model raises questions about madness versus normality within the social order of mental health services. The participating mental health professionals refer to a dichotomy between professionals and service users, and that this dichotomy is the backdrop for stigma and exclusion—and, we would argue, the backdrop for the experienced social drama manifested by mental health professionals who have lived experiences as mental health service users.

## Conclusion

The social order within mental health services does not favor mental health professionals sharing lived experiences of being service users themselves when they are not employed as peer support workers. The results indicated that the participants’ lived experiences influenced their professional practices and social roles in different ways, manifesting in a social drama in which they tried to solve the crisis of a threatened professionalism when sharing their lived experiences within the current social order. Being “betwixt and between” social roles, the participants were in a liminal state in which they either became *keepers of the social order,* who integrated their lived experiences into the role of an expert by either sharing their experiences in ways that supported treatment or by camouflaging their lived experiences as expert knowledge, thereby not disturbing the social order significantly, or they became *violators of the social order,* challenging the social order or separating themselves from it. They fought for a new social role by breaking the boundaries of permitted ways of sharing lived experiences or by preventing the recurrence of negative experiences from their own time spent as mental health service users. Further, analysis indicated that the participants’ social drama resulted in *reintegration,* whereby their lived experiences were, to a degree, acknowledged as relevant within the current social order, even though a full reintegration process in which they could share all their lived experiences was not possible, or a breakdown or *schismogenesis*, resulting from the participants questioning their employments.

Based on these results, we propose a comprehensive *conceptual model* in which we perceive the crisis of mental health professionals who are trying to integrate lived experiences into their daily practices as a social drama.

We invite researchers and stakeholders to be curious about the transformative potential of lived experiences of all mental health professionals in influencing practices within mental health services and recognize the unique challenges these professionals are facing. Mental health professionals with lived experiences as mental health service users have the potential to challenge the current social order and the daily practices of mental health services. They may also challenge the notion of who is defined as “mad” or “sane”—and whether this dichotomy is even relevant.
